# An Evaluation of the Effects of Pineapple-Extract and Bromelain-Based Treatment after Mandibular Third Molar Surgery: A Randomized Three-Arm Clinical Study

**DOI:** 10.3390/nu16060784

**Published:** 2024-03-09

**Authors:** Alessandro Colletti, Chiara Procchio, Mariaelena Pisano, Alma Martelli, Marzia Pellizzato, Giancarlo Cravotto

**Affiliations:** 1 Department of Drug Science and Technology, University of Turin, 10125 Turin, Italy; 2 Italian Society of Nutraceutical Formulators (SIFNut), 31033 Treviso, Italy; alma.martelli@unipi.it (A.M.); pellizzato@recercare.com (M.P.); 3 Studio Dentistico Pisano Procchio, 15121 Alessandria, Italy; procchiara@hotmail.com (C.P.); mariaelena_pisano2003@yahoo.it (M.P.); 4 Department of Pharmacy, University of Pisa, 56126 Pisa, Italy; 5 Interdepartmental Research Centre “Nutraceuticals and Food for Health (NUTRAFOOD)”, University of Pisa, 56120 Pisa, Italy; 6 Interdepartmental Research Centre of Ageing, Biology and Pathology, University of Pisa, 56120 Pisa, Italy

**Keywords:** *Ananas comosus* by-products, bromelain, nutraceutical, freeze-dried juice, third molar surgery, analgesic, anti-inflammatory

## Abstract

A three-arm, randomized, placebo-controlled clinical study was conducted to assess the impact of lyophilized pineapple extract with titrated bromelain (Brome-Inf^®^) and purified bromelain on pain, swelling, trismus, and quality of life (QoL) following the surgical extraction of the mandibular third molars. Furthermore, this study examined the need for Non-Steroidal Anti-Inflammatory Drugs (NSAIDs) by comparing their effects with a placebo group. This study enrolled 42 individuals requiring the extraction of a single mandibular third molar under local anesthesia. The patients were randomly assigned to receive Brome-Inf^®^, purified bromelain, or a placebo orally, initiating treatment on the day of surgery and continuing for the next 7 days. The primary outcome measured was the requirement for NSAIDs in the three groups. Pain, swelling, and trismus were secondary outcome variables, evaluated postoperatively at 1, 3, and 7 days. This study also assessed the comparative efficacy of freeze-dried pineapple extract and single-component bromelain. Ultimately, the placebo group showed a statistically higher need for ibuprofen (from days 1 to 7) at the study’s conclusion (*p* < 0.0001). In addition, reductions in pain and swelling were significantly higher in both the bromelain and pineapple groups (*p* < 0.0001 for almost all patients, at all intervals) than in the placebo group. The active groups also demonstrated a significant difference in QoL compared to the placebo group (*p* < 0.001). A non-significant reduction in trismus occurred in the treatment groups compared to the placebo group. Therefore, the administration of pineapple extract titrated in bromelain showed significant analgesic and anti-edema effects in addition to improving QoL in the postoperative period for patients who had undergone mandibular third molar surgery. Moreover, both bromelain and Brome-Inf^®^ supplementation reduced the need for ibuprofen to comparable extents, proving that they are good alternatives to NSAIDs in making the postoperative course more comfortable for these patients. A further investigation with larger samples is necessary to assess the pain-relieving and anti-inflammatory impacts of the entire pineapple phytocomplex in surgical procedures aside from mandibular third molar surgery.

## 1. Introduction

The proteolytic complex extracted from pineapples (*Ananas comosus*), called “bromelain”, is well known to possess anti-inflammatory, anti-edema, and analgesic properties, which indicates that it may be prescribed for several conditions characterized by the presence of acute inflammation, with or without edema [1]. Bromelain consists of cysteine endopeptidases, which act by catalyzing the hydrolysis of peptide bonds in amino acids that are not at the terminal positions [2]. Although this enzymatic complex’s mechanisms of action are not fully understood, several in vitro and in vivo studies have underlined three targets of action: first, fibrinolytic activity, which proceeds via the activation of factor XI and the modulation of the kallikrein–kinin pathway; second, the regulation of the arachidonic cascade and the production of inflammatory cytokines; and third, the limitation of neutrophil migration to inflammation sites [3]. These actions permit bromelain from being potentially effective in several conditions, as has been highlighted in several randomized clinical trials (RCTs), which have demonstrated the anti-inflammatory, analgesic, and anti-edema activities of bromelain in rheumatoid arthritis, perioperative sport injuries, osteoarthritis, chronic rhinosinusitis, cardiovascular diseases, and skin burns and wounds [4]. In this regard, bromelain may also contribute to reducing the inflammation and edema caused by oral surgery. A recent meta-analysis involving six RCTs has shown that bromelain effectively reduces postoperative pain seven days after mandibular third molar surgery (*p* = 0.002) and diminishes facial swelling in both the early and late postoperative stages (*p* = 0.02 and *p* = 0.0004, respectively) [5]. Comparable results have been reported in previous meta-analyses performed by Mendes et al. [6], de Almeida et al. [7], and de Souza et al. [8], the last of which also showed improvements in sleep quality and social isolation.

Ibuprofen is the most frequently prescribed analgesic/anti-inflammatory drug in dental surgery, followed by naproxen and acetaminophen [9]. However, although Non-Steroidal Anti-Inflammatory Drugs (NSAIDs) spontaneously resolve inflammation, generally within a week, this conventional therapy is not free from side effects [10]. In fact, a percentage of patients have reported excessive NSAID dosing, and, although ibuprofen doses under 1200 mg/day only marginally elevate the risk of gastrointestinal bleeding, the prescribed dose significantly amplifies this risk (relative risk of 4 vs. no medication) [11]. The risk is more pronounced with prolonged usage. However, one study has indicated that patients commencing naproxen face higher risks than those starting ibuprofen, and this difference becomes apparent within 14 days [12]. This suggests that even a few days of use results in increased potential for injury. Some studies have estimated that up to 15,000 people die annually from complications related to NSAID treatment in the United States [13], and their overuse is a potential major health issue.

In recent years, clinical studies have shown that oral supplementation with nutraceuticals may help to reduce inflammation, pain, and/or edema in subjects with chronic inflammatory diseases, reducing the need for NSAIDs [3]. Brome-Inf^®^ is one of these nutraceutical substances, and it is a freeze-dried extract of pineapple, highly concentrated in bioactive peptides and bromelain, and marketable as a food supplement or functional food. Numerous clinical studies have explored the use of bromelain for managing pain and inflammation linked to impacted third molar surgery. These studies have consistently shown decreases in the doses and frequency of conventional anti-inflammatory/analgesic drug administration [7]. To our knowledge, this study represents the first attempt to compare the activity of the pineapple phytocomplex (titrated in 8% bromelain) with purified bromelain. Moreover, bromelain in the form of freeze-dried pineapple is also a functional food with good palatability.

The aim of this study is to investigate the potential role of oral nutraceutical supplementation in people subjected to mandibular third molar surgery in order to reduce the need for NSAIDs and improve quality of life. The second objective is to assess variations in effectiveness between freeze-dried pineapple extract and single-component bromelain.

## 2. Materials and Methods

### 2.1. Study Design and Participants

**Figure 1:**
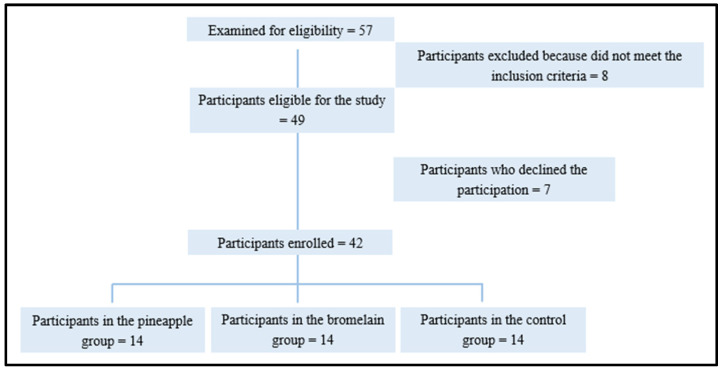
Flowchart of participants in the study (eligible or not eligible; participants excluded for not meeting inclusion criteria = 8; eligible or not eligible; patients who declined participation = 7).

This was a pilot, three-arm, double-blind randomized study. It involved patients enrolled for third molar surgery who were randomized to a ratio of 1:1:1 (Figure 1) to receive pineapple extract, purified bromelain, or a placebo for 7 days after surgery. The study population included 42 healthy individuals belonging to the “Studio Dentistico Pisano Procchio” of Alessandria, who required third molar surgery under local anesthesia. The inclusion criteria were as follows: participants aged between 18 and 35 years, in good health, with a partial bony impacted mandibular third molar, devoid of pericoronitis and infection at the time of surgery, having abstained from medication in the preceding two weeks, and not possessing a history of allergy to the drugs used in the trial. Exclusion criteria encompassed the presence of comorbidities or any medical or surgical conditions that could complicate or compromise the patient’s adherence to the study protocol. Additionally, exclusion criteria included concurrent use of other supplements, allergies, or intolerances to the active ingredient or excipients. Patients were excluded from this study if they had incomplete data or missed scheduled visits, or if they reported the use of non-trial drugs during the observation period.

**Figure 2:**
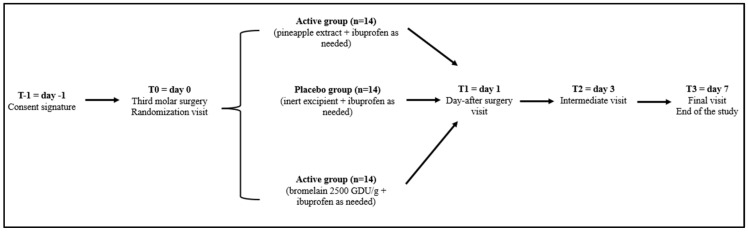
Study timeline.

Informed consent was obtained (T = −1) the day before surgery, and participants were then randomized to receive pineapple extract, purified bromelain, or placebo for 7 days. On T = 0 (day of surgery), T = 1 (day 1), T = 2 (day 3), and T = 3 (day 7), patients were evaluated for clinical status, in addition to being evaluated for compliance and the tolerability of the products. The study’s timeline is described in detail in Figure 2.

### 2.2. Treatment

After they signed the consent form (T−1), at the time of randomization (T0), each patient was treated with Brome-Inf^®^ (freeze-dried pineapple powder containing 200 mg of bromelain in every 2.5 g of powder, measured with a spoon), bromelain (200 mg of bromelain 2500 GDU/g in every 2.5 g of powder, measured with a spoon), or placebo (similar in taste and shape) to be taken orally; 2.5 g was administered every 6 h starting from the morning of surgery and for 3 days after (T2), and 2.5 g was administered every 12 h for the following 4 days (T3). Subjects were instructed to take 600 mg of ibuprofen as needed if pain became significant (for a maximum of t.i.d.). Moreover, postoperatively, all patients in the study received amoxicillin + clavulanic acid (1 tablet t.i.d. equal to 3 g/day) for 5 days after surgery.

Throughout the entire study duration, patients were directed to take the designated treatment at approximately the same time each day, ideally on an empty stomach. Clinical examinations were conducted on days 1, 3, and 7 after surgeries to assess pain, swelling, and trismus. On day 4 after surgery, patients were provided with a quality of life (QoL) questionnaire to complete, and they returned for suture removal by day 7. Additionally, the total count of rescue analgesic tablets consumed during this period was documented.

The study products were manufactured and packaged by Studio 3 Farma srl (Torre di Mosto, Italy) in accordance with Quality Management System ISO 9001:2015 [14].

Centralized randomization was conducted through computer-generated codes. Both participants and investigators were kept blinded to group assignments. The alphanumeric codes (X, Y, and Z) for randomization were kept closed inside an envelope that was kept in a locked drawer in the main investigator’s desk. It was opened by the principal investigator at the end of the study.

### 2.3. Product Preparation

In this work, we scaled up a previously reported lab-scale process [15]. Thoroughly washed, size-selected pineapples (*Ananas comosus* L.) at a uniform ripening stage were cut into fruit slices (rings) using the GINACA–TFGK-5, a processing machine obtained from Tropical Food Machinery srl (Busseto, Italy). This automatic cylinder-forming machine produces cored cylinders from calibrated pineapple fruit. It has an automated and continuous system for loading, transporting, and centering the fruit, as well as a processing group that facilitates peeling, cutting, and coring. The surplus pineapple core and external pulp were reclaimed for juice production, while the remaining peels were expelled. Subsequently, the cored cylinders and pulp were promptly chilled to 4 °C, mechanically blended using a pilot-scale blender (Waring Commercial, Stamford, CT, USA), and subjected to centrifugation at 5000× *g* and 4 °C (Beckman Instruments, Palo Alto, CA, USA), effectively separating insoluble particles from the juice. Dry matter analysis was conducted for 50 g of juice, which was dried to a constant weight at 105 °C overnight in a laboratory dry oven, in adherence to the established standards outlined in the AOAC method 922.10. The resulting residue was quantified and reported as a percentage of the initial material. The outcomes are expressed as grams of solid matter per 100 g of fresh pineapple. Subsequently, the juice underwent rapid cooling in liquid nitrogen and was subjected to freeze-drying using a Criofarma C560-12 unit followed by fast packaging under vacuum.

**Figure 3:**
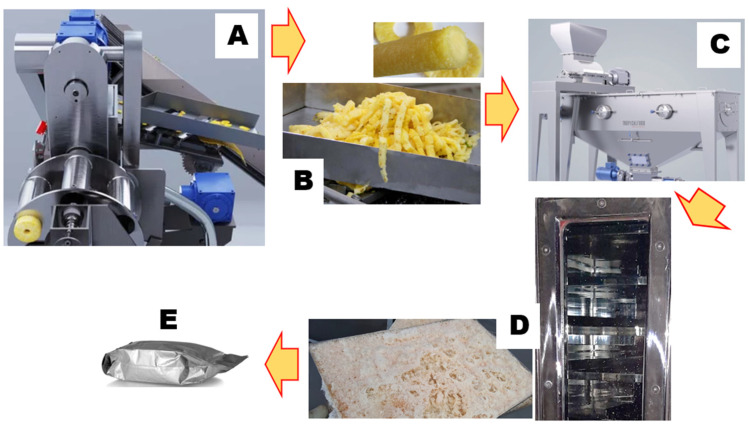
(**A**) Pineapple processing. (**B**) Core and pulp waste. (**C**) Continuous single-body press; (**D**) Industrial freeze-drying. (**E**) Industrial packaging.

The scheme depicted in Figure 3 summarizes the Brome-Inf^®^ preparation steps, from the byproduct to the final product, using a strategy aiming toward a circular economy.

### 2.4. Efficacy Assessment

The primary endpoint of the study aimed to compare the necessity for ibuprofen consumption among the three groups. Key outcome variables encompassed postoperative evaluations of pain, swelling, trismus, and QoL scores recorded following the surgery. Postoperative pain was measured using a 10 cm Visual Analog Scale (VAS), ranging from 0 for ‘no pain’ to 10 for ‘the worst possible pain’. Facial swelling on the operative side was assessed through two facial measurements: tragus–pogonion and gonion–lateral canthus. The sum of these two values (in millimeters) before surgery served as the baseline for that side. Trismus was quantified by determining the difference in interincisal distance at maximal mouth opening before and after the surgery.

The impact on quality of life (QoL) was evaluated using a questionnaire that has been thoroughly described and validated in a previously published report [16]. The questionnaire comprises several items addressing aspects such as social isolation, working isolation, eating ability and dietary variation, speaking ability, sleep impairment, and physical appearance. Recovery for each quality of life (QoL) item was defined using a 4-point scale, with responses categorized as follows: not at all (coded 0), little (coded 1), quite a lot (coded 2), and very much (coded 3). The total score ranged from 0 to 42, with higher scores indicating poorer QoL. Other outcome variables encompassed demographic factors, including age, gender, and body mass index (BMI). Intraoperative variables included the duration of surgery (measured in minutes from the incision to the last suture), while postoperative variables involved the number of rescue analgesic tablets taken by patients up to day 7.

### 2.5. Assessment of Safety and Tolerability

Tolerability and safety were assessed using continuous monitoring over the study period to detect any adverse events and evaluate the clinical safety of the treatment. Treatment compliance and the occurrence of adverse effects were monitored using a diary sheet organized on tables with the possibility for patients to indicate their assumptions of whether they were undergoing nutraceutical or placebo treatment, their eventual ibuprofen intake and number of administrations, and side effects.

### 2.6. Statistical Analysis

Personal data and physiological/pathological anamnesis were only collected at the enrolment visit (T − 1), and treatment compliance data were only collected in T3. The sample size was determined to achieve a power of 80%, with a significance level of 0.05, for a specified difference in pain recorded at a mean of 1 cm on the Visual Analog Scale (VAS). A required sample size of 14 patients per group was identified as necessary to facilitate a statistical model analysis of the differences among the study groups.

Data were systematically entered into an electronic sheet (Excel 2023, Microsoft 2023, Windows 2003, Redmond, WA, USA) throughout the study period. The entries underwent a double check for errors and were subsequently processed using GraphPad Prism 8.0.2 software for Windows. A descriptive analysis was conducted for each variable. The demographic and clinical characteristics of the patients were analyzed using one-way analysis of variance (ANOVA), followed by Bonferroni’s test. A significance level of <0.05 was deemed statistically significant for all conducted tests.

## 3. Results

Forty-nine individuals who required the extraction of a single mandibular third molar under local anesthesia and met all the inclusion criteria were initially enrolled. However, seven patients were subsequently excluded due to non-attendance at follow-up visits or the use of non-study drugs. Therefore, the final analysis included 42 patients who attended follow-up visits and completed the questionnaire. The average age of the participants, comprising 19 men and 23 women, was 22.8 years, with a range of 19 to 27. No statistically significant differences were observed in the demographic characteristics of the subjects or in parameters related to the surgical procedure among the study groups (Table 1), except for the BMI of the patients assigned to the bromelain group, which was found to be significantly lower than the BMI of the placebo group (*p* = 0.0002).

Regarding perceived pain, a significant reduction in pain (VAS-10) was observed in the Brome-inf^®^ and bromelain groups compared to the placebo group (*p* < 0.0001 for both, at all intervals) (Table 2). In addition, the patients in the Brome-inf^®^ and bromelain groups reported approximately half the average intake of ibuprofen compared to the placebo group (*p* < 0.0001 for both active groups).

The highest swelling measurements were reported 1 day after surgery in all study groups (Table 2). The disparity in swelling magnitude between the bromelain group and the placebo group was statistically significant (*p* = 0.037) on day 1. Furthermore, a comparison among the groups indicated a substantial reduction in swelling on days 3 and 7 in both the bromelain and Brome-Inf^®^ groups (*p* < 0.0001) as opposed to the placebo group.

The mean baseline measurements of interincisal distance were 46, 44, and 45 mm in the placebo, bromelain, and Brome-Inf^®^ groups, respectively. Trismus reached its maximum in all groups one day after surgery and subsequently subsided at the subsequent follow-up intervals. However, a comparative analysis of the groups did not reveal any statistically significant differences (Table 2).

As regards the QoL measurements, both active groups demonstrated significant reductions in scores in all areas (social, work, eating, sleep, speech, and appearance) compared with the placebo group (Table 3). A significant improvement was also seen in the total QoL score for both the bromelain and Brome-Inf^®^ groups compared to the placebo group (placebo vs. bromelain *p* = 0.0001; placebo vs. Brome-Inf^®^ *p* = 0.0002).

No side effects were reported during the treatment. Moreover, both Brome-Inf^®^ and bromelain supplementation showed good palatability and excellent compliance (100%). No cases of alveolar osteitis or wound infection were reported during the study period.

## 4. Discussion

Over the past few decades, a new healthcare paradigm emphasizing diet and nutrition has gained prominence. A health-conscious consumer base with greater disposable income in the Western world has redirected consumer trends toward the acquisition of dietary supplements, functional foods, and nutraceuticals. The aim is to sustain optimal health, prevent chronic pathologies that impact quality of life, and enhance overall lifespan [17]. Epidemiological studies suggest that there exists an association between the consumption of nutraceuticals and the prevention of several diseases [18].

The nutraceutical market presently stands as a thriving multi-billion-euro industry, garnering a remarkable global response. Its estimated value was around USD 383 billion in 2016, with projections anticipating a growth to approximately USD 561 billion by 2022, prior to the onset of the coronavirus (COVID-19) pandemic [19]. In addition, the value of the nutraceuticals industry is already more than 25% of the value of the pharmaceutical industry [19].

One of the most interesting nutraceuticals, bromelain, is widely used for the prevention or co-management of numerous diseases that are characterized by the presence of inflammation, edema, and algesia. Although several clinical trials have demonstrated the efficacy of bromelain supplementation in the reduction in pain [20], inflammation [21], and edematous components [22], its commercial cost is high, while the isolation and purification of bromelain from pineapple (fruit, core, stems, and leaves) is an open issue, making up 70–90% of the final extract production cost [23]. Moreover, notwithstanding the advent of novel and viable protein purification methods such as membrane filtration, reverse micellar systems, aqueous two-phase extraction, and chromatographic techniques, as well as the development of new biotechnological processes aimed at reducing production costs, various limitations persist. These challenges continue to impact the efficiency of product recovery from crude plant extracts and the overall effectiveness of the obtained extract. The enzyme complex tends to undergo irreversible inactivation at elevated temperatures, as encountered in processes like pasteurization. Concurrently, the gradual concentration of bromelain in crude pineapple juice throughout the purification process has the potential to induce spontaneous enzymatic deactivation [3]. In this context, the use of freeze-dried pineapple juice extract obtained from by-products (core and peel of *Ananas comosus*), which respects the concepts of “zero waste approach” and the “circular economy”, has been shown to preserve a good quantity of total bromelain (up to 8% of dry weight) in its active form.

This study delves into the impact of lyophilized pineapple extract, both titrated and standardized in bromelain, as well as purified bromelain on postoperative outcomes and measures of QoL following the surgical extraction of impacted mandibular third molars. Our study, based on the work by Majid et al. [24], demonstrates that the oral intake of bromelain in multiple daily doses, starting on the day of surgery and continuing for 7 days, resulted in a significant effect on the clinical and QoL statuses of these patients. In particular, the regular assumption of bromelain, both as a functional food and in its purified form (200 mg every 6 h starting on the morning of surgery and continuing for 3 days after, and 200 mg every 12 h for the following 4 days), has been observed to significantly reduce ibuprofen intake compared with that of the placebo group, acting as a painkiller and inflammation treatment. In this regard, previous studies have demonstrated that the effects of bromelain are comparable to those of pre-emptive diclofenac sodium or ibuprofen in the third molar surgery setting [6,7,8]. Moreover, both groups (pineapple and bromelain) were observed to have a positive effect on QoL measurements after third molar removal, with this likely being due to their anti-edema, anti-inflammatory, and analgesic effects. Moreover, they display excellent safety profiles (no adverse reaction reported) and good palatability. In this context, the pineapple extract and bromelain study groups showed a marked antiphlogistic effect, which was higher than that of the placebo group (characterized by the statistically higher consumption of ibuprofen). Ibuprofen was selected as the reference drug in this study to serve as a representative of the NSAIDs family. As anticipated, it demonstrated a noteworthy analgesic and anti-inflammatory effect during the initial postoperative period in the placebo group.

Bromelain has demonstrated therapeutic advantages even at doses as modest as 160 mg/day. However, it is commonly believed that optimal results are achieved for most conditions with doses ranging from 750 to 1000 mg/day, administered in four divided doses [3], which was the regimen used in this study. Although the mechanisms of action of bromelain are not yet fully elucidated, it seems to function by removing cell-surface molecules such as CD128a/CXCR1, CD128b/CXCR2, CD16, CD14, CD44, and CD21. These molecules play crucial roles in leukocyte trafficking, cellular adhesion, and the induction of pro-inflammatory mediators, and they exert immunomodulatory effects on T cells. Bromelain also regulates proinflammatory prostaglandins by inhibiting thromboxane A2 and prostaglandin E. Additionally, it modulates p-selectin-mediated neutrophil recruitment [25] and regulates the blood levels of bradykinin and also plasma fibrinogen [26]. These mechanisms make bromelain potentially effective against various conditions associated with inflammation. This justifies its consideration as a potential alternative to NSAIDs.

Various risk factors for edema, pain, and trismus following third molar surgery have been identified by different researchers, including age, gender, operative time, and surgical experience [27]. To mitigate the impact of bias from these factors on our results, treatment allocation was randomized, and strict inclusion criteria were enforced. Furthermore, the surgical procedures were consistently carried out by the same surgeon in all cases to minimize potential operator variability. The implementation of double-blinding also helped overcome any potential personal bias from both the patients and the surgeon [28].

This study also demonstrates that the supplements provide a significant improvement in QoL, highlighting that pineapple extract may be an adjuvant to help improve QoL in individuals subjected to third molar surgery. Moreover, to the best of our knowledge, this is the first study to investigate the effect of the entire phytocomplex of a lyophilized pineapple by-product on the QoL status of patients after oral surgery and compare the effects with bromelain as a single component. This may be particularly important, especially in the context of a circular economy; starting from the waste products from the pineapple food chain, it was possible to obtain a particularly effective titrated and standardized extract, adopting the so-called “zero waste approach”. In this regard, one of the most relevant aspects of this study concerns the overlap in the results obtained from the pineapple extract and the single component bromelain. Although the dosages of bromelain were comparable in the two active groups, the purified bromelain exhibited superior enzymatic activity (2500 GDU/g vs. 400 GDU/g of pineapple extract). Consequently, it is important to consider whether the evaluation of the enzymatic activity through the measurement of the GDU is a predictive method for in vivo effects and, above all, whether the impact of the entire phytocomplex is to be preferred over the single protease mixture. Indeed, multiple studies have indicated that the pharmacological effects of bromelain are only partially linked to its proteolytic activity. This highlights the significance of assessing the entire phytocomplex, which includes non-protein factors, for a comprehensive understanding of its effects [29]. These aspects require extensive future research.

Our study was limited by the small number of participants involved. However, it highlights the need for further large-scale RCTs to examine the analgesic and anti-edema efficacy of pineapple extract and purified bromelain. Larger and more extensive studies are also still needed to verify the scalability in the production of pineapple extracts from food industry by-products; to analyze the final cost of the raw material in industrial production and to conduct a detailed analysis into the cost/benefit ratio of this nutraceutical; to evaluate, in in vitro studies, the active ingredients present in the freeze-dried pineapple phytocomplex that may have an additive effect on bromelain; to study the pharmacokinetics of bromelain, which are almost completely unknown; and to evaluate the long-term efficacy and safety profile of bromelain (even at high doses) before considering the prescription of this nutraceutical in clinical practice.

## 5. Conclusions

In conclusion, the administration of pineapple extract containing a daily oral dose of 800 mg of bromelain for the first 3 days and 400 mg for the following 4 days and, separately, the same dosages of purified bromelain, showed significant analgesic and antiedema effects in addition to improving the QoL for patients who had undergone mandibular third molar surgery. Moreover, both pineapple and bromelain supplementation reduced the need for NSAIDs, demonstrating that this treatment is a possible alternative to ibuprofen and that it provides a more comfortable postoperative course for patients. Further research with larger samples is essential to assess the analgesic and anti-inflammatory effects of the entire phytocomplex of pineapple in surgical procedures beyond third molar surgery.

## Figures and Tables

**Table 1:** Patient demographics and intraoperative parameters.

Variable	Bromelain (*n* = 14)	Brome-Inf^®^ (*n* = 14)	Placebo (*n* = 14)	Total	*p* Value
**Age (year)**	22.4 ± 4.9	22.9 ± 4.5	23.1 ± 4.1	22.8 ± 4.5	n.s.
**Gender**					
***Male***	5	6	8	19	n.a.
***Female***	8	7	8	23	n.a.
**BMI (Kg/m^2^)**	24.4 ± 0.2 ***	24.6 ± 0.2	24.8 ± 0.3	24.6 ± 0.2	Placebo vs. Bromelain *p* = 0.0002 Placebo vs. Brome-Inf^®^ *p* = 0.0963 n.s. Bromelain vs. Brome-Inf^®^ *p* = 0.0963 n.s.
**Operation time (min)**	31.2 ± 14.1	32.7 ± 18.2	31.5 ± 17.4	31.8 ± 16.5	n.s.

n.s., not significant; n.a., not applicable; *** *p* < 0.001 compared vs. placebo; data presented as mean ± standard deviation. BMI: body mass index.

**Table 2:** Comparison of outcome variables among and within study groups.

Variable	Placebo (*n* = 14)	Bromelain (*n* = 14)	Brome-Inf^®^ (*n* = 14)	*p* Value
**VAS-10**				
*Day-1*	3.857 ± 0.462	2.286 ± 0.529 ****	2.121 ± 0.387 ****	Placebo vs. Bromelain *p* < 0.0001 Placebo vs. Brome-Inf^®^ *p* < 0.0001 Bromelain vs. Brome-Inf^®^ *p* > 0.9999, n.s.
*Day-3*	2.836 ± 0.325	1.514 ± 0.419 ****	1.457 ± 0.238 ****	Placebo vs. Bromelain *p* < 0.0001 Placebo vs. Brome-Inf^®^ *p* < 0.0001 Bromelain vs. Brome-Inf^®^ *p* > 0.9999, n.s.
*Day-7*	1.629 ± 0.190	0.407 ± 0.144 ****	0.450 ± 0.129 ****	Placebo vs. Bromelain *p* < 0.0001 Placebo vs. Brome-Inf^®^ *p* < 0.0001 Bromelain vs. Brome-Inf^®^ *p* > 0.9999, n.s.
	D1 vs. D3; D3 vs. D7; D1 vs. D7: *p* < 0.0001 ^§§§§^)	(D1 vs. D3; D3 vs. D7; D1 vs. D7: *p* < 0.0001 ^§§§§^)	(D1 vs. D3; D3 vs. D7; D1 vs. D7: *p* < 0.0001 ^§§§§^)	
**Swelling**				
*Day-1*	8.193 ± 0.329	7.7779 ± 0.345 **	7.929 ± 0.264	Placebo vs. Bromelain *p* = 0.0037 Placebo vs. Brome-Inf^®^ *p* = 0.0969, n.s. Bromelain vs. Brome-Inf^®^ *p* = 0.6440, n.s.
*Day-3*	4.236 ± 0.448	3.093 ± 0.329 ****	3.257 ± 0.253 ****	Placebo vs. Bromelain *p* < 0.0001 Placebo vs. Brome-Inf^®^ *p* < 0.0001 Bromelain vs. Brome-Inf^®^ *p* = 0.6775, n.s.
*Day-7*	1.843 ± 0.214	1.207 ± 0.219 ****	1.243 ± 0.320 ****	Placebo vs. Bromelain *p* < 0.0001 Placebo vs. Brome-Inf^®^ *p* < 0.0001 Bromelain vs. Brome-Inf^®^ *p* > 0.9999, n.s.
	D1 vs. D3; D3 vs. D7; D1 vs. D7: *p* < 0.0001 ^§§§§^	D1 vs. D3; D3 vs. D7; D1 vs. D7: *p* < 0.0001 ^§§§§^	D1 vs. D3; D3 vs. D7; D1 vs. D7: *p* < 0.0001 ^§§§§^	
**Trismus**				
*Day-1*	13.1 ± 3.7	13.4 ± 3.9	13.1 ± 3.7	Placebo vs. Bromelain *p* > 0.9999, n.s. Placebo vs. Brome-Inf^®^ *p* > 0.9999, n.s. Bromelain vs. Brome-Inf^®^ *p* > 0.9999, n.s.
*Day-3*	8.9 ± 2.1	7.5 ± 2.3	7.8 ± 2.5	Placebo vs. Bromelain *p* = 0.3488, n.s. Placebo vs. Brome-Inf^®^ *p* = 0.6431, n.s. Bromelain vs. Brome-Inf^®^ *p* > 0.9999, n.s.
*Day-7*	5.4 ± 1.5	4.3 ± 1.1	4.6 ± 1.2	Placebo vs. Bromelain *p* = 0.0850, n.s. Placebo vs. Brome-Inf^®^ *p* = 0.3171 Bromelain vs. Brome-Inf^®^ *p* > 0.9999, n.s.
	D1 vs. D3: *p* = 0.0004 ^§§§^ D3 vs. D7: *p* = 0.0030 ^§§^ D1 vs. D7: *p* < 0.0001 ^§§§§^	D1 vs. D3: *p* < 0.0001 ^§§§§^ D3 vs. D7: *p* = 0.0095 ^§§^ D1 vs. D7: *p* < 0.0001 ^§§§§^	D1 vs. D3: *p* < 0.0001 ^§§§§^ D3 vs. D7: *p* = 0.0089 ^§§^ D1 vs. D7: *p* < 0.0001 ^§§§§^	
**Rescue tablets of ibuprofen**	6.4 ± 1.4	3.6 ± 1.2 ****	3.2 ± 1.4 ****	Placebo vs. Bromelain *p* < 0.0001 Placebo vs. Brome-Inf^®^ *p* < 0.0001 Bromelain vs. Brome-Inf^®^ *p* > 0.9999 n.s.

** *p* < 0.01; **** *p* < 0.0001 compared with placebo; ^§§^ *p* < 0.01; ^§§§^ *p* < 0.001; ^§§§§^ *p* < 0.0001 compared between days; n.s. not significant. Data presented as mean ± standard deviation. VAS: Visual Analogue Scale.

**Table 3:** Comparison of outcome variables among and within study groups.

Variable	Placebo (*n* = 14)	Bromelain (*n* = 14)	Brome-Inf^®^ (*n* = 14)	*p* Value
Social	0.9 ± 0.3	0.4 ± 0.2 ****	0.3 ± 0.2 ****	Placebo vs. Bromelain: *p* < 0.0001 Placebo vs. Brome-Inf^®^: *p* < 0.0001 Bromelain vs. Brome-Inf^®^: *p* = 0.8196 n.s.
Work	1.1 ± 0.4	0.6 ± 0.4 **	0.4 ± 0.3 ****	Placebo vs. Bromelain: *p* = 0.0028 Placebo vs. Brome-Inf^®^: *p* < 0.0001 Bromelain vs. Brome-Inf^®^: *p* = 0.4809, n.s.
Eating	8.1 ± 1.7	5.7 ± 1.0 ****	5.8 ± 0.9 ****	Placebo vs. Bromelain: *p* < 0.0001 Placebo vs. Brome-Inf^®^: *p* < 0.0001 Bromelain vs. Brome-Inf^®^: *p* > 0.9999, n.s.
Speech	1.8 ± 0.9	1.1 ± 0.7 ****	1.2 ± 0.6 ****	Placebo vs. Bromelain: *p* < 0.0001 Placebo vs. Brome-Inf^®^: *p* < 0.0001 Bromelain vs. Brome-Inf^®^: *p* > 0.9999, n.s.
Sleep	2.4 ± 1.1	0.7 ± 0.5 ****	0.9 ± 0.4 ****	Placebo vs. Bromelain: *p* < 0.0001 Placebo vs. Brome-Inf^®^: *p* < 0.0001 Bromelain vs. Brome-Inf^®^: *p* > 0.9999, n.s.
Appearance	2.9 ± 1.2	1.2 ± 0.7 ****	1.5 ± 0.8 ***	Placebo vs. Bromelain: *p* < 0.0001 Placebo vs. Brome-Inf^®^: *p* = 0.0008 Bromelain vs. Brome-Inf^®^: *p* > 0.9999, n.s.
Total	17.2 ±5.6	9.7 ± 3.5 ***	10.1 ± 3.2 ***	Placebo vs. Bromelain: *p* = 0.0001 Placebo vs. Brome-Inf^®^: *p* = 0.0002 Bromelain vs. Brome-Inf^®^: *p* > 0.9999, n.s.

** *p* < 0.01; *** *p* < 0.001; **** *p* < 0.0001 compared with placebo; n.s. not significant. Data presented as mean ± standard deviation.

## Data Availability

Data is contained within the article.
